# Etiology and early clinical predictors of neurological outcomes in pediatric spontaneous intracranial hemorrhage: a retrospective exploratory study

**DOI:** 10.3389/fneur.2025.1750475

**Published:** 2026-01-08

**Authors:** Wei Hou, Shengjuan Wang, Jiangshun Fang

**Affiliations:** Department of Pediatric Neurosurgery, Hebei Children’s Hospital, Shijiazhuang, China

**Keywords:** etiology, Glasgow Coma Scale, modified Rankin Scale, pediatric spontaneous intracranial hemorrhage, prognostic factors, seizure

## Abstract

**Background:**

Pediatric spontaneous intracranial hemorrhage (sICH) is rare but clinically diverse, and prognostic evaluation remains challenging because current models rely mainly on anatomical severity rather than etiologic or early clinical features. This study aimed to characterize the etiologic spectrum of pediatric sICH and identify early predictors of 3-month neurological outcomes.

**Methods:**

This retrospective study included children aged 1 month to 14 years diagnosed with sICH at Hebei Children’s Hospital from December 2016 to December 2024. Patients were divided into four etiologic groups: unknown causes, vascular causes, blood-related causes, and other defined causes. Clinical, radiologic, and laboratory data at admission were collected. Neurological outcomes were assessed using the modified Rankin Scale (mRS) at 3 months, with poor outcome defined as mRS > 2. Variables significant in univariate analyses (*p* < 0.1) were entered into multivariate logistic regression to identify independent predictors of poor outcome.

**Results:**

Among 148 children (median age 48.0 months; 39.2% female), vascular (35.1%) and unknown etiologies (33.8%) were most common. Poor outcomes occurred in 58 patients (39.2%). In multivariate analysis, seizures at onset (OR = 2.861, 95% CI: 1.076–7.612, *p* = 0.035) and other defined etiologies—including infections, tumors, and systemic diseases—were strong independent predictors of poor recovery (OR = 8.025, 95% CI: 1.606–40.112, *p* = 0.011). Vomiting at presentation emerged as a novel protective factor (OR = 0.292, 95% CI: 0.112–0.763, *p* = 0.012); these findings were exploratory and require further validation. Higher admission Glasgow Coma Scale (GCS) scores were also protective (OR = 0.795, 95% CI: 0.667–0.946, *p* = 0.010).

**Conclusion:**

The etiologic distribution of pediatric sICH is markedly diverse, and the prognosis at 3 months is substantially influenced by both etiology and early clinical characteristics. Seizures at onset and secondary etiologies (such as infections and tumors) significantly increase the risk of poor outcome, whereas vomiting and higher GCS scores are associated with more favorable recovery. Early integration of etiologic classification and clinical presentation may enhance prognostic accuracy and guide individualized management strategies in pediatric sICH.

## 1 Introduction

Spontaneous intracranial hemorrhage (sICH) is a rare but critical neurological emergency in children (1, 2). Despite its low overall incidence, the risk of death and long-term neurological complications remains high (3, 4). Survivors frequently suffer from permanent neurological deficits, which limit their daily functional independence and place a significant economic, emotional, and social burden on their families (5–7). With the widespread use and advancement of imaging technology, the survival rate and overall prognosis of children with sICH have improved (8, 9). However, due to the varied etiology, the cause of hemorrhage in some children remains unknown even after extensive imaging and laboratory examinations, highlighting the limitations of current diagnostic techniques and the incomplete understanding of the disease pathophysiology (10–12).

Pediatric sICH is highly variable due to the interplay of clinical and imaging factors. Previous research has shown that hematoma volume and location, and altered mental status are important independent predictors of neurological outcome (7, 13). Clinical scoring systems, such as the Pediatric Intracerebral Hemorrhage (PICH) score and the modified PICH (mPICH) score, have been developed for risk classification in sICH among children (13, 14). These models have substantial therapeutic utility, however, they are primarily concerned with anatomical and physiological variables, leaving out etiological and clinical elements that may have a significant impact on prognosis. Combining etiological classification with early clinical features can improve the accuracy and clinical utility of prognostic predictions, allowing for the timely identification of high-risk children (7, 14, 15). This is critical for making clinical judgments about neurosurgical procedures and intensive care monitoring (4). Furthermore, survivors may require long-term multidisciplinary care, which includes neurosurgery follow-up, rehabilitation, and cognitive support, emphasizing the importance of treatment planning and customized prognostic assessment (6). Understanding how etiology and early clinical factors influence prognosis is critical for informing treatment decisions and developing public health policies (2, 3, 16).

Our study sought to define the etiological spectrum of pediatric sICH and investigate the association between early clinically related features and neurological outcomes. We used an eight-year cohort to examine how underlying causes and initial presentation affect prognosis. These findings aim to support risk stratification and guide evidence-based clinical management (3).

## 2 Materials and methods

### 2.1 Study design and participants

This study was conducted in accordance with the ethical guidelines of the Declaration of Helsinki. Informed consent was obtained from the parents or legal guardians of all patients. Patient confidentiality was maintained by anonymizing all data. The study protocol was approved by the Institutional Review Board of Hebei Children’s Hospital (No. 2024115). This retrospective cohort study included sICH admitted to the Neurosurgery Department of Hebei Children’s Hospital between December 2016 and December 2024. Eligible patients were aged 28 days to 14 years and had computed tomography (CT) or magnetic resonance imaging (MRI) confirming intracranial hemorrhage. This age limitation reflects our hospital’s patient distribution, as children beyond the age of 14 were seldom admitted and would not provide an appropriate sample size for credible statistical analysis. Only non-traumatic cases were included, and all patients had normal intelligence and motor development before onset.

The exclusion criteria were:

1. Hemorrhage secondary to head trauma;
2. Incomplete or severely missing clinical data;
3. Death within 24 h of admission;
4. Failure to complete laboratory or imaging studies required to clarify the etiology.

**Figure 1:**
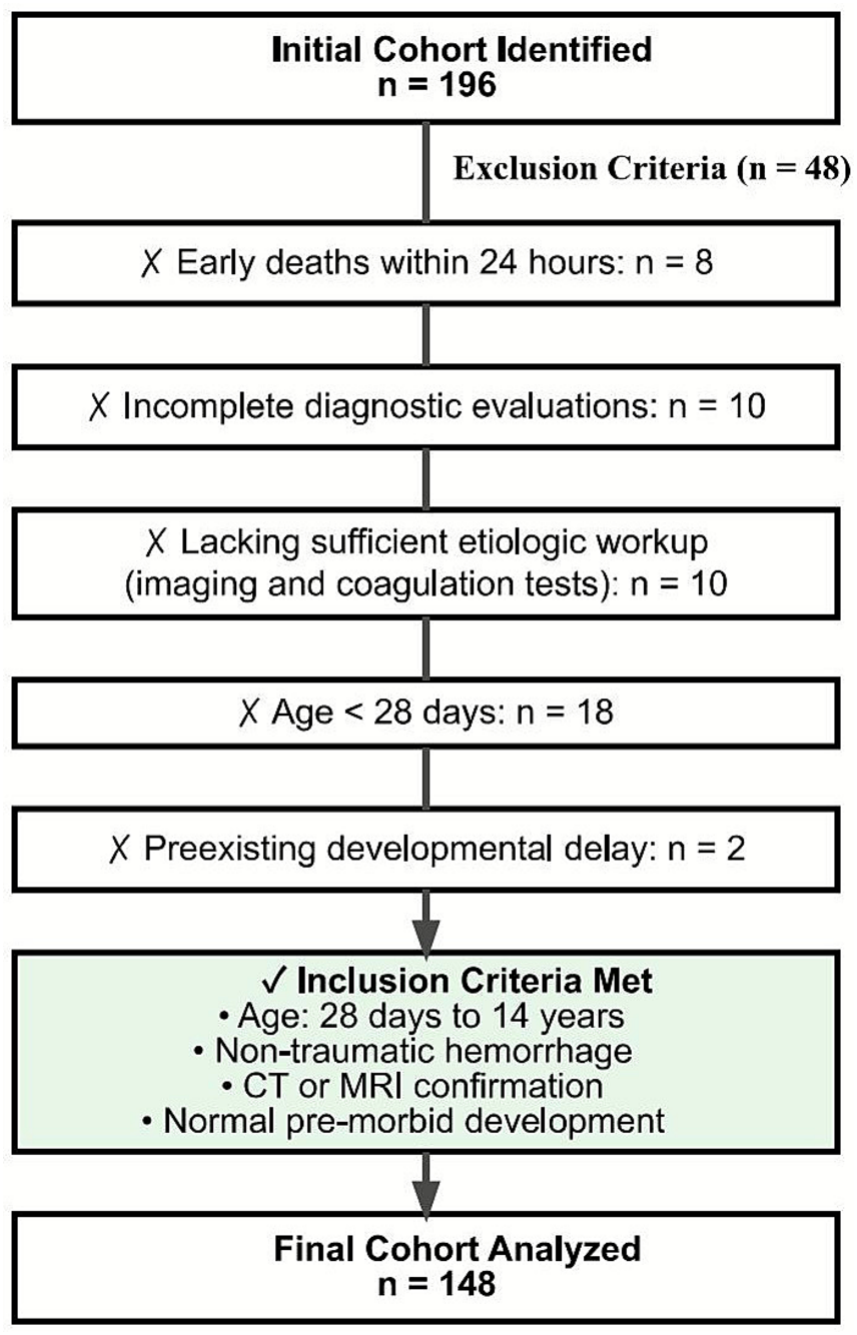
Patient selection process for spontaneous intracranial hemorrhage study. CT, computed tomography; MRI, magnetic resonance imaging.

Among 196 patients screened, 48 were excluded (8 early deaths, 10 incomplete diagnostic evaluations, 10 lacking sufficient etiologic workup such as imaging and coagulation-related examinations, 18 infants <28 days, and 2 with preexisting developmental delay), leaving 148 patients for final analysis (Figure 1).

To ensure accurate etiologic classification, patients assigned to the unknown cause were required to have undergone adequate diagnostic evaluation to exclude vascular, hematologic, and infectious causes. This assessment typically included CT or MRI, noninvasive vascular imaging such as magnetic resonance angiography (MRA) and magnetic resonance venography (MRV), and baseline laboratory tests including complete blood count, coagulation profile, and inflammatory markers. Digital subtraction angiography (DSA) was selectively performed when noninvasive imaging suggested a possible vascular abnormality or when vascular etiology could not be excluded, as determined by the treating neurosurgical team.

### 2.2 Data collection

Clinical data, including demographic information, clinical manifestations, imaging results, laboratory results, and treatment details, were all obtained from the hospital’s electronic medical record system. Two pediatric neurosurgeons independently reviewed and cross-validated all data to ensure their completeness and accuracy.

Clinical characteristics included age, gender, a history of premature birth and hypoxia, time from symptom onset to admission, and presenting symptoms such as vomiting, headache, seizure, and loss of consciousness. The admission Glasgow Coma Scale (GCS) score was used to determine the patient’s initial neurological condition.

Radiologic information obtained from CT or MRI included the location and type of bleeding, hematoma volume (estimated using the ABC/2 method), intraventricular extension, hydrocephalus, and evidence of brain herniation.

Laboratory variables included white blood cell count (WBC), hemoglobin (Hb), platelet count (PLT), prothrombin time (PT), activated partial thromboplastin time (APTT), international normalized ratio (INR), and fibrinogen (FIB).

Complications during hospitalization included anemia, electrolyte abnormalities, pneumonia, hepatic or renal dysfunction, cardiac damage, hydrocephalus, and symptomatic epilepsy.

The etiology of hemorrhage was determined based on imaging findings, laboratory evidence, and clinical evaluation, and was categorized into four groups: (1) vascular causes, (2) blood-related causes, (3) other defined causes, and (4) unknown causes. Vascular causes were confirmed by DSA, computed tomography angiography (CTA), MRI, MRA/MRV, or postoperative pathological examination. Hematologic causes were identified based on clinical history and coagulation-related laboratory tests. Other defined causes were diagnosed through imaging, cerebrospinal fluid analysis, microbiological testing, or clinical records. Cases were classified as unknown when no etiology could be determined after comprehensive clinical, imaging, and laboratory evaluation.

### 2.3 Outcome measures

Neurological outcomes were assessed using the modified Rankin Scale (mRS) 3 months after discharge. Favorable outcome was defined as mRS ≤ 2, and poor outcome as mRS > 2. Three-month mRS scores were obtained from outpatient follow-up records or structured telephone interviews documented in the electronic medical system. For retrospective cases in which an explicit mRS score was not recorded, two trained pediatric neurologists independently reviewed clinical descriptions of functional status and assigned mRS scores using a standardized protocol. Any discrepancies were resolved through discussion with a senior neurologist. Post-hemorrhagic epilepsy (PHE) was also evaluated as an additional outcome measure. PHE was defined as any unprovoked seizure occurring after the acute hemorrhagic event, confirmed by clinical observation and/or electroencephalography (EEG) during hospitalization or follow-up. Mortality referred to in-hospital or follow-up death. Recurrence was defined as new hemorrhage confirmed by imaging during hospitalization or follow-up.

### 2.4 Statistical analysis

All statistical analyses were performed using SPSS version 26.0 (IBM Corp., Armonk, NY, USA). Continuous variables were tested for normality using the Shapiro–Wilk test and reported as mean ± standard deviation (SD) or median (interquartile range, IQR), as appropriate; categorical variables were summarized as counts and percentages. Comparisons between the favorable and poor outcome groups were conducted using the independent-samples *t*-test or the Mann–Whitney U test for continuous variables and the chi-square test or Fisher’s exact test for categorical variables. These group-comparison tests provided the *p* values shown in the corresponding tables. To further evaluate the association between each variable and poor outcome, univariable logistic regression analyses were performed to obtain unadjusted odds ratio (OR) and 95% confidence interval (CI). Variables with *p* < 0.1 in univariable logistic regression, along with clinically important factors (e.g., etiology and GCS), were entered into the multivariable logistic regression model to identify independent predictors of poor outcome. For categorical variables, the unknown causes group was used as the reference category in all logistic regression analyses, as it represents the etiologic subgroup without a defined pathological process and provides a neutral baseline for comparison. To evaluate potential multicollinearity among predictors included in the multivariate logistic regression model, variance inflation factors (VIFs) were calculated using linear regression with all candidate variables entered simultaneously. ORs and 95% CIs were calculated. A *p* value < 0.05 was considered statistically significant.

## 3 Results

**Figure 2:**
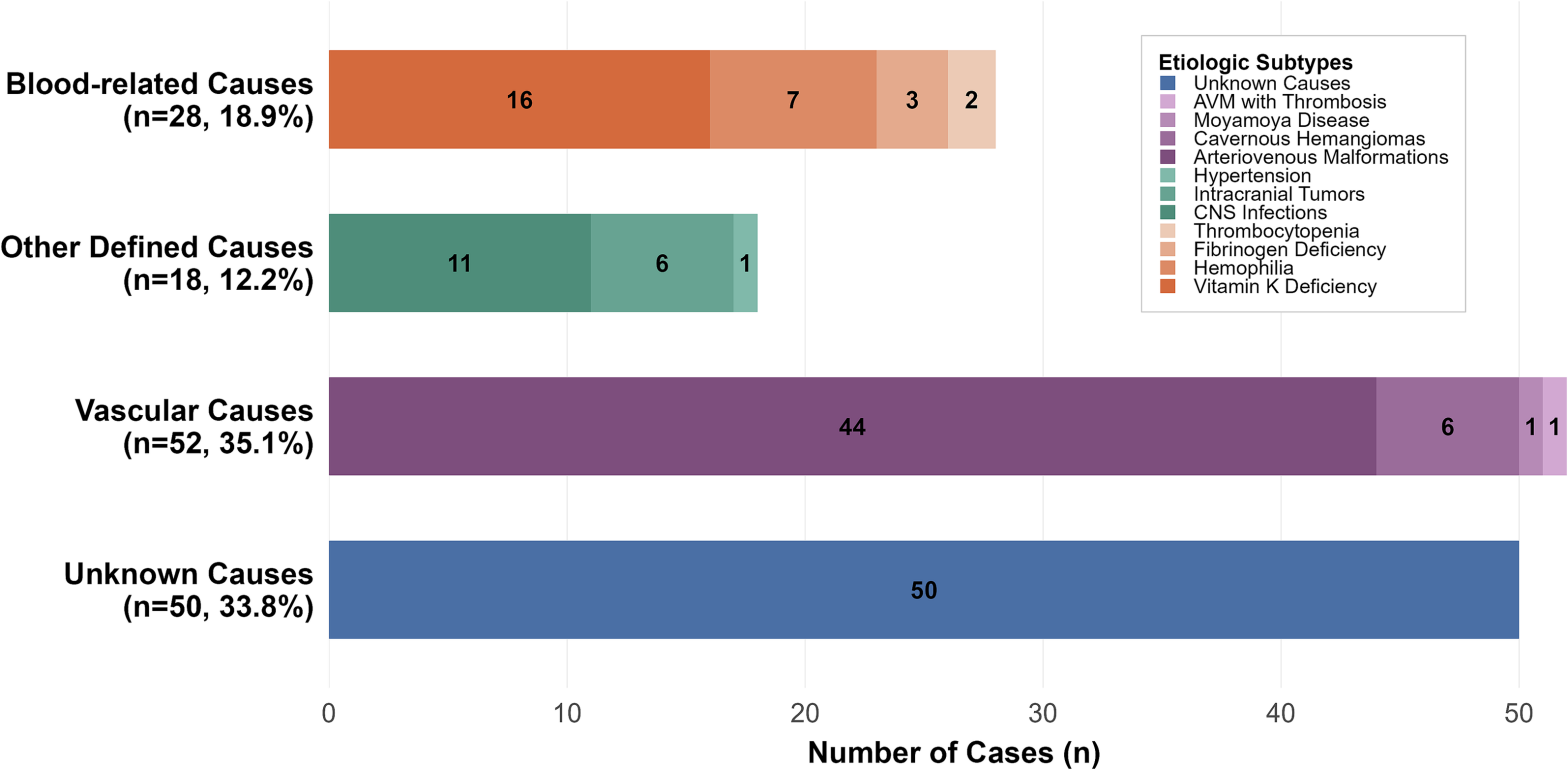
The etiologic distribution of spontaneous intracranial hemorrhage. AVM, arteriovenous malformation; CNS, central nervous system.

In all, 148 children with sICH were enrolled. Patients were divided into four etiological groups: unknown causes (*n* = 50), vascular causes (*n* = 52), other defined causes (*n* = 18), and blood-related causes (*n* = 28) (Figure 2). Vascular causes accounted for the majority of sICH cases (*n* = 52, 35.1%), including arteriovenous malformations (AVMs, *n* = 44), cavernous hemangiomas (*n* = 6), Moyamoya syndrome, and one AVM with thrombosis. Hematologic disorders were identified in 28 patients (18.9%), primarily due to vitamin K deficiency (*n* = 16), hemophilia (*n* = 7), fibrinogen deficiency (*n* = 3), and thrombocytopenia (*n* = 2). Other defined causes (*n* = 18, 12.2%) comprised central nervous system infections (*n* = 11), intracranial tumors (*n* = 6) and one case secondary to hypertension. The remaining 50 cases (33.8%) had no identifiable etiology despite comprehensive evaluation and were classified as idiopathic or cryptogenic.

### 3.1 Baseline characteristics and etiologic distribution

The age distribution varied markedly among etiologic groups, but the gender difference was not significant (Table 1). Children with vascular malformations were the oldest, with a median age of 84 months (IQR 60–120), while those with blood-related diseases were the youngest, with a median age of only 2.0 months (IQR 1.5–4.0). The unknown etiology group had a median age of 30 months, and children with other defined causes were mostly infants and toddlers (median 18.0 months). These findings indicate distinct age-related etiologic patterns.

**Table 1:** Baseline characteristics by etiology in pediatric spontaneous intracranial hemorrhage.

Characteristics	Total (*n* = 148)	Unknown causes (*n* = 50)	Vascular causes (*n* = 52)	Other defined causes (*n* = 18)	Blood-related causes (*n* = 28)
Sex (Female), *n* (%)	58 (39.2)	19 (38.0)	22 (42.3)	8 (44.4)	9 (32.1)
Age(Months), median (IQR)	48.0 (2.19, 96.0)	30.0 (2.0, 120.0)	84.0 (60.0, 120.0)	18.0 (7.3, 36.0)	2.0 (1.5, 4.0)
Initial symptom, *n* (%)					
Vomiting	63 (42.6)	24 (48.0)	24 (46.2)	5 (27.8)	10 (35.7)
Headache	47 (31.8)	17 (34.0)	25 (48.1)	3 (16.7)	2 (7.1)
Seizure	45 (30.4)	15 (30.0)	12 (23.1)	6 (33.3)	12 (42.9)
Time to presentation, *n* (%)					
<24 h	71 (48.0)	26 (52.0)	30 (57.7)	2 (11.1)	13 (46.4)
24 h–72 h	27 (18.2)	14 (28.0)	4 (7.7)	1 (5.6)	8 (28.6)
>72 h	50 (33.8)	10 (20.0)	18 (34.6)	15 (83.3)	7 (25.0)
Site of hemorrhage, *n* (%)					
Lobe	87 (58.8)	24 (48.0)	35 (67.3)	10 (55.6)	18 (64.3)
Subdural	33 (23.3)	12 (24.0)	4 (7.7)	5 (27.8)	12 (42.9)
Deep brain	41 (27.7)	20 (40.0)	12 (23.1)	2 (11.1)	7 (25.0)
Cerebellum	13 (8.8)	5 (10.0)	6(11.5)	0 (0.0)	2 (7.1)
IVH	63 (42.6)	22 (44.0)	25 (48.1)	6 (33.3)	10 (35.7)
Multiple sites	35 (23.6)	7 (14.0)	9 (17.3)	3 (16.7)	16 (57.1)
Hematoma volume, *n* (%)					
<10 ml	58 (39.2)	23 (46.0)	16 (30.8)	12 (66.7)	7 (25.0)
10 ml–30 ml	35 (23.6)	16 (32.0)	13 (25.0)	0 (0.0)	6 (21.4)
>30 mL	55 (37.2)	11 (20.0)	23 (44.2)	6 (33.3)	15 (53.6)
Herniation, *n* (%)	42 (28.4)	8 (16.0)	21 (40.4)	4 (22.2)	9 (32.1)
GCS on admission, median (IQR)	10.5 (8.0, 13.0)	12.0 (9.0, 14.0)	10.0 (8.0, 14.0)	11.5 (8.0, 13.0)	10.0 (8.5, 12.5)
Surgical intervention, *n* (%)	89 (61.0)	21 (43.8)	49 (94.2)	9 (50.0)	10 (35.7)
Complication, *n* (%)	98 (66.2)	28 (56.0)	27 (51.9)	16 (88.9)	29 (96.7)

IQR, interquartile range; IVH, intraventricular hemorrhage; GCS, Glasgow Coma Scale.

Initial symptoms also differed by cause. Vomiting was the most common presenting feature (42.6%), particularly in unknown (48.0%) and vascular (46.2%) cases. Headache occurred most frequently in vascular lesions (48.1%), while seizures were more frequent in blood-related (42.9%) and other defined causes (33.3%) groups. Regarding the interval from symptom onset to hospital presentation, children with other defined causes had the longest delay: 83.3% presented more than 72 h after onset, compared with only 20.0% in the unknown group and 25.0% in the blood-related group. In contrast, over half of vascular cases (57.7%) were admitted within 24 h.

Lobar hemorrhage was the most frequent type overall (58.8%), especially among vascular causes (67.3%) and blood-related causes (64.3%). Deep brain hemorrhage was most common in the unknown group (40.0%), while subdural hematoma was frequent in blood-related disorders (42.9%) and “other defined causes” (27.8%). Multiple bleeding sites occurred in 23.6% of patients, predominantly in the blood-related group (57.1%).

Regarding hematoma volume, one-third (37.2%) of patients had large hemorrhages (>30 mL). Vascular causes accounted for the highest proportion of large hematomas (44.2%), whereas the other defined causes group more often had small volumes (<10 mL, 66.7%). Brain herniation was observed in 42 children (28.4%), again most frequent in vascular (40.4%) and blood-related (32.1%) cases.

The median GCS score on admission was 10.5 (IQR 8–13). Children with vascular causes had relatively lower initial GCS (median 10.0), while the unknown group showed slightly higher scores (median 12.0). Surgical intervention was performed in 89 patients (61.0%), nearly universal in vascular cases (94.2%) but less common in other defined causes (50.0%) and blood-related causes (35.7%). Complications occurred in 98 children (66.2%). The incidence of complications was highest in blood-related (96.7%) and other defined causes (88.9%), and lowest in vascular (51.9%) and unknown etiologies (56.0%).

### 3.2 Comparative and univariate analysis of factors associated with neurological outcomes

Univariate analysis identified several factors associated with neurological outcomes in pediatric sICH (Table 2). Etiology showed a significant impact (*p* = 0.022). Compared with the unknown-cause group, children with other defined causes had markedly higher odds of poor outcomes (61.1%; OR = 4.976, 95% CI: 1.578–15.693, *p* = 0.006), followed by blood-related disorders, which demonstrated a trend toward association (46.4%; OR = 2.744, 95% CI: 1.024–7.359, *p* = 0.045). Vascular causes showed no significant association (*p* = 0.332). Younger age was linked to worse prognosis (median 12.0 vs. 62.0 months, *p* = 0.032; OR = 0.989, 95% CI: 0.982–0.996), whereas sex and perinatal factors showed no differences. Regarding initial symptoms, vomiting (49.5% vs. 30.2%, *p* = 0.023; OR = 0.442, 95% CI: 0.217–0.899) and headache (41.1% vs. 15.1%, *p* = 0.001; OR = 0.255, 95% CI: 0.108–0.601) were associated with favorable outcomes. Conversely, seizures at onset strongly predicted poor prognosis (49.1% vs. 20.0%, *p* < 0.001; OR = 3.852, 95% CI: 1.844–8.047). Time from symptom onset to presentation showed no significant differences between outcome groups (*p* = 0.955). Approximately one-third of children in each group presented after 72 h, suggesting that delayed admission did not independently influence prognosis in this cohort. Hospital length of stay was also similar between groups (median 18.5 vs. 19.0 days, *p* = 0.749). Radiologic findings also differed between groups. Poor outcomes were associated with deep brain hemorrhage (39.6% vs. 21.1%, *p* = 0.016; OR = 2.461, 95% CI: 1.175–5.153), larger hematoma volume (*p* = 0.002), and brain herniation (39.6% vs. 22.1%, *p* = 0.023; OR = 1.233, 95% CI: 1.027–1.481). Admission GCS scores were significantly lower in the poor-outcome group (median 9.0 vs. 12.0, *p* < 0.001; OR = 0.793, 95% CI: 0.704–0.893). Lower hemoglobin levels were significantly associated with poor outcomes (*p* = 0.001; OR = 0.963, 95% CI: 0.969–0.997), while reduced fibrinogen showed a borderline association (*p* = 0.045; OR = 0.617, 95% CI: 0.378–1.007). No other laboratory parameters, including white blood cell count, platelet count, or coagulation indices, were significantly associated with prognosis. Surgical intervention was also not significantly associated with outcome (*p* = 0.664). Complications were more frequent in patients with poor prognosis (83.0% vs. 56.8%, *p* = 0.001).

**Table 2:** Comparison of clinical, radiologic, and laboratory characteristics between favorable and poor outcome groups.

Causes	Favorable outcome (*n* = 90)	Poor outcome (*n* = 58)	*p* (group comparison)	Unadjusted OR (95% CI)
Etiology, *n* (%)			0.022	
Unknown causes	38 (76.0)	12 (24.0)	—	1.000
Vascular causes	35 (67.3)	17 (32.7)	0.332	1.538 (0.644, 3.671)
Other defined causes	7 (38.9)	11 (61.1)	0.006	4.976 (1.578, 15.693)
Blood-related causes	15 (53.6)	13 (46.4)	0.045	2.744 (1.024, 7.359)
Sex (Female), *n* (%)	36 (37.9)	22 (41.5)	0.666	1.163 (0.586, 2.309)
Age (Months), median (IQR)	62.0 (2.17, 120.0)	12.0 (2.18, 72.0)	0.032	0.989 (0.982, 0.996)
Premature birth, *n* (%)	10 (10.5)	4 (7.5)	0.916	1.441 (0.429, 4.841)
History of hypoxia, *n* (%)	4 (4.2)	2 (4.8)	0.997	0.892 (0.158, 5.041)
Initial symptom, *n* (%)				
Vomiting	47 (49.5)	16 (30.2)	0.023	0.442 (0.217, 0.899)
Headache	39 (41.1)	8 (15.1)	0.001	0.255 (0.108, 0.601)
Seizure	19 (20.0)	26 (49.1)	0.000	3.852 (1.844, 8.047)
Time to presentation, *n* (%)			0.955	0.982 (0.674, 1.432)
<24 h	45 (47.4)	26 (49.1)		
24 h–72 h	18 (18.9)	9 (17.0)		
>72 h	32 (33.7)	18 (34.0)		
Length of stay, median (IQR)	19.0 (16.0, 24.0)	18.5 (12.5, 24.0)	0.749	1.012 (0.988, 1.036)
Site of hemorrhage, *n* (%)				
Intracerebral	54 (56.8)	33 (62.3)	0.522	1.253 (0.630, 2.493)
Subdural	23 (24.2)	10 (8.9)	0.456	0.728 (0.317, 1.675)
Deep brain	20 (21.1)	21 (39.6)	0.016	2.461 (1.175, 5.153)
Cerebellum	10 (10.5)	3 (5.2)	0.218	0.510 (0.134, 1.941)
IVH	41 (43.2)	22 (41.5)	0.846	0.935 (0.473, 1.846)
Multiple sites	19 (20.0)	16 (30.2)	0.163	1.147 (0.945, 1.391)
Hematoma volume, *n* (%)			0.002	1.423 (0.963, 2.104)
<10 ml	38 (40.0)	20 (37.7)		
10 ml–30 ml	30 (31.6)	5 (9.4)		
>30 ml	27 (28.4)	28 (52.8)		
Herniation, *n* (%)	21 (22.1)	21 (39.6)	0.023	1.233 (1.027, 1.481)
GCS on admission, median (IQR)	12.0 (9.0, 14.0)	9.0 (6.0, 11.5)	0.000	0.793 (0.704, 0.893)
WBC	13.35 ± 5.26	13.59 ± 5.10	0.577	1.009 (0.969, 0.997)
Hb	116.78 ± 24.17	104.94 ± 33.24	0.001	0.963 (0.969, 0.997)
Plt	350.04 ± 132.17	338.92 ± 156.45	0.474	0.999 (0.946, 1.077)
PT	14.01 ± 13.14	16.28 ± 24.06	0.957	1.007 (0.988, 1.026)
APTT	34.24 ± 24.99	38.40 ± 31.61	0.934	1.005 (0.993, 1.017)
INR	1.12 ± 0.62	1.45 ± 2.30	0.888	1.172 (0.895, 1.535)
FIB	2.28 ± 0.93	1.98 ± 0.72	0.045	0.617 (0.378, 1.007)
Surgical intervention, *n* (%)	58 (62.4)	31 (58.5)	0.664	1.176 (0.591, 2.342)
Complication, *n* (%)	54 (56.8)	44 (83.0)	0.001	0.269(0.118, 0.614)

*p*-values were derived from *χ*^2^, Fisher’s exact test, or Mann–Whitney U test as appropriate. Odds ratios and 95% confidence intervals were calculated using univariate logistic regression.

Units: WBC (×10^9^/L), Hb (g/L), Plt (×10^9^/L), PT (s), APTT (s), INR (ratio), FIB (g/L).

OR, odds ratio; CI, confidence interval; IQR, interquartile range; IVH, intraventricular hemorrhage; GCS, Glasgow Coma Scale; WBC, White Blood Cells Hb, hemoglobin; Plt, platelet count; PT, prothrombin time; APTT, activated partial thromboplastin time; INR, international normalized ratio; FIB, fibrinogen.

Pediatric reference ranges are age-dependent. The approximate ranges used in this study are as follows: WBC, 4.5–17.5 × 10^9^/L (varies with age); Hb, 110–140 g/L (varies with age); Plt, 150–400 × 10^9^/L; PT, 11–14 s; APTT, 25–40 s; INR, 0.9–1.2; FIB, 1.5–4.0 g/L.

In general, poorer outcomes were related to younger age, seizures at onset, deep brain involvement, large hematoma volume, low GCS, and decreased hemoglobin/fibrinogen. A milder clinical presentation and a more favorable prognosis were indicated by vomiting and headache at onset.

### 3.3 Clinical outcomes across etiologic groups

Poor outcomes occurred in 35.8% of patients (Table 3). Rates were highest in other defined causes (61.1%) and blood-related etiologies (46.4%), intermediate in vascular lesions (32.7%), and lowest in the unknown group (24.0%). Symptomatic epilepsy was uncommon (6.8%) and showed no major differences among etiologies. Thirteen children (8.8%) died, with mortality concentrated in blood-related (17.9%) and other defined causes (16.7%), lower in vascular lesions (9.6%), and absent in the unknown group. Recurrent hemorrhage occurred in 6.1% of patients and was most frequent in vascular causes (9.6%), with only minor variations among the remaining groups. Overall, secondary and hematologic etiologies carried the greatest burden of adverse outcomes, whereas recurrence and epilepsy rates were relatively similar across groups.

**Table 3:** Neurological outcomes, mortality, and recurrence according to etiology.

Variable	Total (*n* = 148)	Unknown causes (*n* = 50)	Vascular causes (*n* = 52)	Other defined causes (*n* = 18)	Blood-related causes (*n* = 28)
Poor outcome	53 (35.8)	12 (24.0)	17 (32.7)	11 (61.1)	13 (46.4)
Symptomatic epilepsy	10 (6.8)	2 (4.0)	3 (5.8)	2 (11.1)	3 (10.7)
Mortality	13 (8.8)	0 (0.0)	5 (9.6)	3 (16.7)	5 (17.9)
Recurrent hemorrhage	9 (6.1)	1 (2.0)	5 (9.6)	1 (5.6)	2 (7.1)

### 3.4 Multivariate analysis

**Figure 3:**
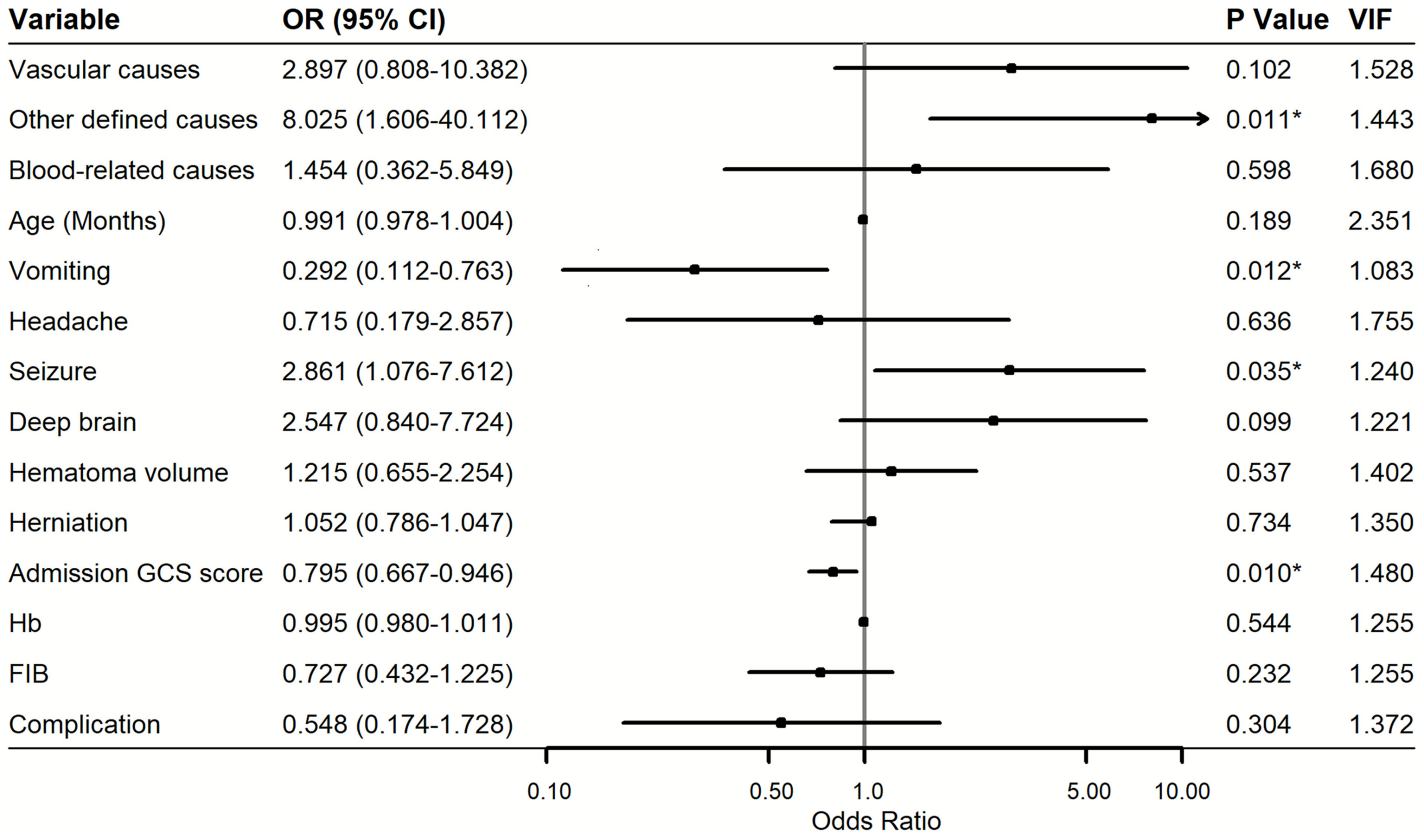
Multivariable logistic regression analysis for predictors of poor outcome. OR, odds ratio; CI, confidence interval; GCS, Glasgow Coma Scale; Hb, hemoglobin; FIB, fibrinogen. Reference group: unknown causes. *p < 0.001.

Variables with *p* < 0.1 in univariate analysis were included in the multivariate model (Figure 3). The reference group for etiologic comparisons was the unknown-cause group. Independent predictors of poor neurological outcome included other defined causes (OR = 8.025, 95% CI 1.606–40.112, *p* = 0.011) and seizures at onset (OR = 2.861, 95% CI 1.076–7.612, *p* = 0.035). Vomiting remained an independent protective factor (OR = 0.292, 95% CI 0.112–0.763, *p* = 0.012), while a lower admission GCS score predicted worse outcomes (OR = 0.795, 95% CI 0.667–0.946, *p* = 0.010). No significant association was observed for age, headache, deep brain, hematoma volume, herniation, hemoglobin, fibrinogen, or complication after adjustment. Additionally, no multicollinearity was detected among variables in the multivariate model, with VIF values ranging from 1.083 to 2.351.

## 4 Discussion

We described the etiological distribution of pediatric sICH and identified early clinical predictors of neurological sequelae, emphasizing the importance of combining clinical insights with etiological information (3, 7, 10). Within this approach, we first looked at the distribution of underlying causes and their clinical significance. Vascular abnormalities emerged as the major cause in our sample, mostly affecting school-aged children and adolescents (10). Age-related differences in cerebrovascular development and hemodynamic stress may contribute to the higher hemorrhage tendency observed in pediatric AVMs compared with adults (17, 18). This trend has important implications for therapy. Most children with vascular abnormalities require surgery or endovascular treatment to avoid rebleeding, and early, focused intervention is typically associated with better results than those with secondary causes (19). Children with coagulopathies or blood abnormalities, on the other hand, have a greater risk of complications and death. Impaired coagulation and widespread bleeding can expand the hematoma, raise intracranial pressure, and cause subsequent brain damage. Prompt identification and correction of coagulation issues is consequently critical for stabilizing patients and enhancing recovery (11, 20, 21).

Secondary intracerebral hemorrhage caused by factors other than coagulopathies, such as infections, tumors, and hypertension, accounted for 12.2% of cases and was a strong predictor of poor prognosis in multivariate models, demonstrating the clinical importance of recognizing systemic and secondary etiologies in pediatric sICH (10, 11). Although the hemorrhage volume in these children is often small, the primary disease carries a significant systemic burden, and their poor prognosis may reflect the combined effects of the underlying pathology and the hemorrhage itself. In many such patients, neurological deterioration may be primarily caused by the primary disease itself, rather than the size or location of the hemorrhage. Furthermore, symptoms of the primary disease often mask early neurological manifestations, leading to delays in diagnosis and treatment. Given the relatively small number of cases in each subtype, further stratified analysis was not possible; therefore, the observed increased risk should be interpreted as reflecting the combined severity of these systemic etiologies rather than a single hemorrhage mechanism (11, 12). In addition, we found a high proportion of secondary or systemic causes in this study, such as hematologic disorders, infections, and tumors, primarily affecting infants and young children. These causes themselves generally have poor prognoses. Therefore, when interpreting the age-prognosis relationship in spontaneous intracranial hemorrhage in children, it is crucial to consider the heterogeneity of underlying causes, rather than solely relying on age itself (22).

Children with unknown etiology accounted for approximately one-third of the cohort, representing a substantial proportion of pediatric sICH cases. Their outcomes fell between those of the vascular and secondary etiology groups. These children may have hidden vascular malformations or temporary coagulation problems that are difficult to detect with standard imaging (10, 11, 23). In our series, 29 patients (58%) in the unknown group showed cerebrovascular developmental anomalies on MRI. During the follow-up period, two individuals (4%) had clear vascular abnormalities identified. This suggests that many cryptogenic cases involve mild or progressive vascular abnormalities that are missed at first diagnosis. These cases highlight both the limitations of existing imaging techniques and our incomplete understanding of pediatric cerebrovascular development. Future advances in imaging resolution and analytical techniques may help improve diagnostic accuracy and etiologic classification (1, 9, 12).

Further investigation indicated disparities in the location of bleeding between etiologies, and the position of the lesion was strongly associated with prognosis. Deep intracerebral hemorrhages, particularly those involving the basal ganglia and thalamus, were associated with poorer neurological outcomes. This finding is supported by Park and Jang (24). In contrast, lobar and subdural hemorrhage generally indicated a better prognosis (6, 7, 16). Vascular malformations most commonly cause lobar hemorrhage, which has good surgical accessibility and a more favorable prognosis (10, 19). Hematologic disorders cause multi-site bleeding, with lobar hemorrhage combined with subdural hemorrhage being especially common in this group. Patients with multifocal lesions, especially those with hematologic disorders, have a higher incidence of adverse outcomes, with a mortality rate of 17.9%, indicating the impact of diffuse lesions and coagulation disorders (6, 25, 26). We found no association between poor functional outcomes and intraventricular extension or infratentorial, which is consistent with previous findings (27). These imaging patterns yield significant prognostic clues. The anatomical location of the lesion can direct acute-phase treatment, such as decompression for lobar hemorrhage, as well as long-term risk prediction (8, 16).

Vomiting at onset was associated with favorable outcomes in our cohort, a finding not previously reported in pediatric sICH. The underlying mechanism remains unknown, however, several explanations are possible. Vomiting implies that brainstem reflexes are intact, as is some autonomic regulation. Furthermore, vomiting may prompt patients to seek medical attention immediately, allowing for timely treatment before neurological deterioration begins. These characteristics could assist in explaining the relationship between vomiting and a better prognosis. However, these interpretations remain hypothetical, as no prior evidence has demonstrated a protective effect of vomiting in pediatric sICH. Additional multicenter studies are needed to corroborate this discovery and explain its physiological basis. In contrast, early seizures occurred in 30.4% of patients and were independently associated with poor prognosis. This differs from the findings of Williams et al. (28), who reported seizures as protective against in-hospital mortality. The disparity could be attributed to differing study endpoints: we evaluated long-term neurological outcomes, whereas their research focused on short-term survival. Seizures significantly increase brain metabolic demand and oxygen consumption, resulting in secondary hypoxia and worsened neuronal damage. Early seizure management and ongoing EEG monitoring are critical (29–31).

A low GCS score upon admission was consistently related to a poor prognosis, supporting the GCS score’s significant predictive value (2, 3, 22). Combining GCS with clinical and etiologic factors can further improve predictive accuracy, as demonstrated in models such as mPICH (13, 32). Complications and overall systemic status also play a major role in outcomes. Complications occurred in 66.2% of patients and included both neurological and systemic problems. Hydrocephalus occurred in 9.5% of cases, mostly associated with intraventricular extension. Early drainage or shunt procedures were beneficial for recovery (33, 34). Other reversible problems, including anemia, electrolyte imbalance, and pneumonia, typically lengthen hospital stays but are easily managed (22, 26, 29). These complications likely reflect the overall severity of the disease, highlighting the importance of close monitoring. Laboratory test findings can also indicate the body’s systemic stress level and the degree of bleeding. In univariate analysis, lower hemoglobin and fibrinogen levels were related to a worse prognosis; however, in multivariate models, they lost significance, suggesting that they primarily reflect disease severity (35–37). Maintaining adequate hemoglobin and coagulation function remains essential to prevent hematoma expansion.

Mortality in this cohort was 8.8%, which differs from the mortality rate previously reported in prior pediatric ICH studies (6, 7). The difference likely stems from variations in patient mix, inclusion criteria, and follow-up duration. Deaths mainly occurred in children with secondary etiologies, emphasizing that systemic or primary disease rather than hemorrhage volume is often the critical factor (10, 15). No deaths were observed among unknown cases, though this may reflect small sample size or selection bias rather than a truly benign nature. Rebleeding occurred in 6.1% of patients, most within the vascular or hematologic groups. Reports on pediatric sICH recurrence are limited and inconsistent, and no standardized follow-up imaging protocol currently exists (8, 38). Targeted vascular imaging and hematologic monitoring may help reduce recurrence risk. Continuous post-discharge surveillance is essential for these high-risk patients (16).

In our study, symptomatic epilepsy occurred in 6.8% of patients, primarily among those with secondary etiologies. Early cortical irritation may contribute to the development of persistent epilepsy. Persistent epilepsy greatly affects quality of life and long-term rehabilitation, emphasizing the need for early seizure prevention and continuing neurological monitoring. Epilepsy may represent a continuum from acute brain injury to sustained functional impairment. Structured follow-up for high-risk children, including EEG monitoring and neuropsychological examination, may help detect subclinical epileptiform activity before significant functional decline occurs, but direct evidence is lacking (29, 39). Data on post-ICH epilepsy and cognitive outcomes in children remain scarce, and unified models for long-term prognostic prediction are lacking. Large multicenter prospective studies with standardized follow-up are needed to develop robust prognostic models incorporating etiologic, imaging, and neurophysiological measures.

Treatment should be individualized according to etiology. Surgical or endovascular repair of vascular malformations, early correction of coagulopathy, and infection control were all associated with improved outcomes, underscoring the value of etiology-based management (40, 41). Despite these findings, our single-center retrospective design has inherent limitations, including a relatively small sample size and evolving diagnostic resources over the study period. The absence of dynamic laboratory and imaging data limits the assessment of disease progression; ongoing monitoring of hematologic and inflammatory markers may provide better insight into evolving injury (16, 42). Future studies should incorporate enhanced neuroimaging, radiomics, and molecular biomarkers to improve diagnostic accuracy. Machine learning models can help with personalized prognosis and decision-making, resulting in more precise early intervention (43, 44). Establishing a multi-center collaborative database will help overcome sample size constraints and support the development and validation of standardized risk assessment tools for pediatric spontaneous cerebral hemorrhage (45). These efforts will lay a solid platform for more precise and personalized management of children’s sICH.

## 5 Conclusion

This study focuses on the role of etiology and early neurological status in defining the outcomes of children with spontaneous intracranial hemorrhage. Onset seizures, lower GCS scores, and secondary causes such as tumor- or infection-related bleeding were all associated with a poor prognosis, but vomiting appeared to be protective. These findings demonstrate the importance of early etiologic identification, rapid neuroimaging, and timely multidisciplinary intervention. Optimizing early management and referral pathways may improve neurological recovery and survival in affected children. Future multicenter, prospective studies with larger cohorts are warranted to validate these results and explore underlying pathophysiological mechanisms.

## Data Availability

The original contributions presented in the study are included in the article/supplementary material, further inquiries can be directed to the corresponding author.
